# Construction and optimization of a ‘NG Morbidostat’ - An automated continuous-culture device for studying the pathways towards antibiotic resistance in *Neisseria gonorrhoeae*

**DOI:** 10.12688/f1000research.18861.2

**Published:** 2020-01-08

**Authors:** Els Verhoeven, Said Abdellati, Patrick Nys, Jolein Laumen, Irith De Baetselier, Tania Crucitti, Chris Kenyon

**Affiliations:** 1 Department of Clinical Sciences - STI unit, Institute of Tropical Medicine, Antwerp, Antwerp, 2000, Belgium; 2 Department of Clinical sciences - HIV/STI reference laboratory, Institute of Tropical Medicine, Antwerp, Antwerp, 2000, Belgium

**Keywords:** morbidostat, Neisseria gonorrhoeae, antibiotics, macrolide resistance, antimicrobial resistance, sexual transmitted infection

## Abstract

To obtain a detailed picture of the dynamics of antibiotic resistance development in *Neisseria gonorrhoeae*, we built a morbidostat according to the protocol of Toprak *et al*., adjusted to the specific characteristics required for the growth of *N. gonorrhoeae*. In this article we describe the adaptations, specifications and the difficulties we encountered during the construction and optimization of the NG morbidostat. As a proof of concept, we conducted a morbidostat experiment by increasing concentrations of azithromycin in response to bacterial growth. We started the experiment with two *N. gonorrhoeae* reference strains WHO-F and WHO-X. These strains were grown in 12 mL GC Broth supplemented with IsoVitaleX™ (1%) and vancomycin, colistin, nystatin, trimethoprim (VCNT) selective supplement for 30 days in a 6% CO _2_ environment at 36°C. Samples of the cultures were taken 2-3 times a week and minimal inhibitory concentrations (MICs) of azithromycin were determined using E-test. The initial MICs of WHO-F and WHO-X were 0.125 µg/mL and 0.25 µg/mL, respectively. In less than 30 days, we were able to induce high level azithromycin resistance in *N. gonorrhoeae*, with a 750 and 1000 fold increase in MIC for WHO-F and WHO-X, respectively.

## Introduction

Gonorrhoea is a sexual transmitted infection (STI) caused by the obligate human pathogen *Neisseria gonorrhoeae* (gonococcus), a Gram-negative diplococcus ^1, 2^. Transmission of the gonococcus occurs primarily by direct contact between the mucosal membranes in the urogenital tract, anal canal and oropharynx, usually during sexual activity ^2^.

Gonorrhoea is one of the most commonly reported STIs ^3^. The World Health Organization (WHO) estimated an incidence of 87 million new cases for gonorrhoea (individuals aged 15–49 years) in 2016, worldwide ^4^. An increase in cases of gonorrhoea has been reported in several European countries: 11% rise from 2014 to 2015 in the United Kingdom ^5^ and a doubling of cases in 2013 and 2015 among men who have sex with men (MSM) in France ^6^.

There is a growing global concern about antimicrobial resistance in *N. gonorrhoeae*, with resistance reported to almost all antimicrobials previously and currently available for treatment ^7^. In response to this concern, the Centers for Disease Control and Prevention (CDC) and the European guidelines for treatment of gonorrhoea introduced dual antimicrobial therapy with azithromycin (oral) and ceftriaxone (injectable) for uncomplicated gonorrhoea in 2012 ^2, 8^. Dual therapy was also recommended by WHO in 2016 ^9^. However, the prevalence of ceftriaxone and azithromycin-resistant strains is increasing in certain areas ^10, 11^.

Therefore, more research is required to investigate how *N. gonorrhoeae* develops antibiotic resistance so rapidly. A better understanding of the pathways to resistance may enable novel strategies to prevent the emergence of resistance in the future ^12^. In order to evaluate the dynamics of resistance development, Toprak *et al.* ^13^ developed a microbial culture device called a ‘morbidostat’. A morbidostat is a bioreactor that continuously monitors bacterial growth and adjusts antibiotic concentration to induce bacterial resistance against the drug ^13, 14^. Experiments using a morbidostat can give us insights into the evolution of resistance and the nature, order and speed at which mutations arise. Whole-genome sequencing can be used to characterize the sequential mutation steps in the resistance genesis in great detail ^14, 15^.

The aim of this study was to build a morbidostat according to the protocol of Toprak *et al*. ^13^. We report here on the construction, optimization and a proof of concept of the NG morbidostat, a morbidostat adjusted to the specific characteristics required for the growth of *N. gonorrhoeae*.

## Methods

### Principle of the morbidostat

The morbidostat measures growth rates based on the intensity of back-scattered light through a bacterial suspension using an optical detection system. The bacteria are suspended in a fixed volume of liquid medium with continuous stirring. At fixed time intervals, the culture is diluted with a fixed volume. Depending on turbidity measurements and growth rate, an algorithm defines dilution with fresh medium or fresh medium containing antibiotic. Fresh medium with antibiotic is injected if the turbidity exceeds a threshold and when the net growth rate of the bacteria is positive. This threshold is set in the mid-log phase of the growth curve. At this point, there is a high metabolic activity present in the bacteria and the population is not nutrient limited. To allow *N. gonorrhoeae* to adapt to the environment, a threshold for fresh medium is set as well. Over time, the concentration of antibiotic added to these vials increases and drug resistance in bacteria evolves. During the entire experiment, the volume in the morbidostat culture vials is kept constant using a 16-channel suction pump (Ismatec, ISM938D). Morbidostat experiments are continued until a diminishing rate of increase in drug resistance is observed ^13, 14^.

### Construction of the morbidostat

**Figure 1.**
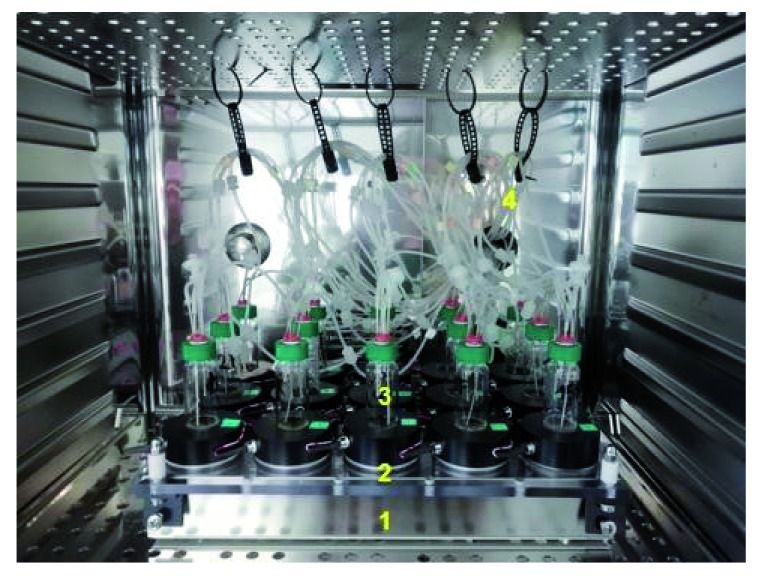
Neisseria gonorrhoeae morbidostat set-up inside the CO _2_ incubator. (1) the magnetic stirrer plate with 15 positions. Upon this, (2) the vial holder array with the optical detection system placed upon the magnetic stirrer plate. (3) morbidostat culture vials that are placed and connected with silicone tubes going through a hole (4) at the back of the incubator to the peristaltic pumps outside the incubator.

**Figure 2.**
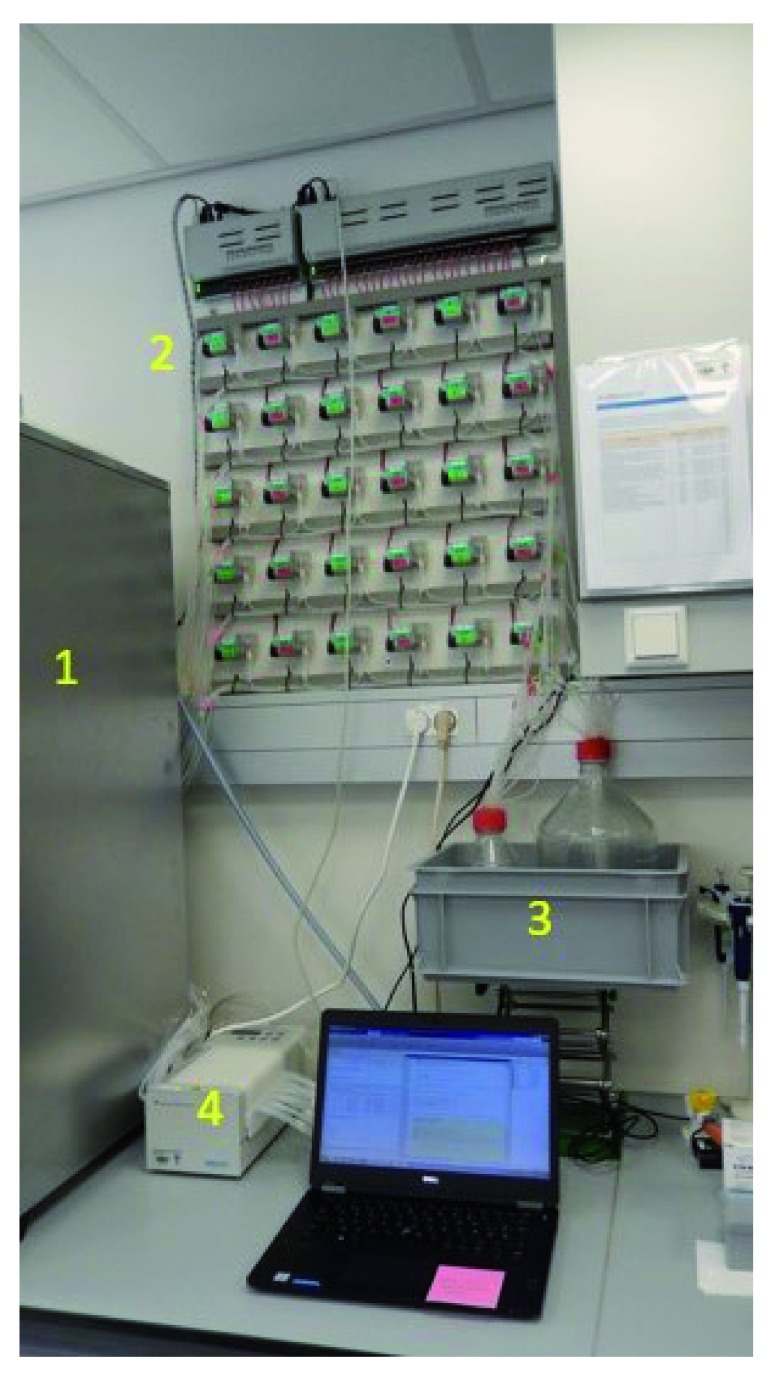
(1) CO _2_ incubator with the culture vials and the vial holder array as shown in Figure 1. Silicone tubes connected to the peristaltic pumps, which are all placed against a plate fixed to the wall (2), (3) reservoirs bottles for plain media and media with antibiotic. (4) The 16-channel suction pump for waste.

The NG morbidostat consisted of two parts: one part containing 15 independent culture vials was built within a CO _2_ incubator ( Figure 1), the other part, outside the incubator, contained the suction pumps and controlling equipment ( Figure 2).

We modified Toprak’s protocol ^13^ and included one drug pump for each culture vial instead of two. This resulted in 30 peristaltic pumps: 15 medium pumps and 15 drug pumps for a capacity of 15 morbidostat culture vials. Consequently, we manually increased the drug concentrations by changing the drug reservoirs after a period of time, usually every three days. This modification made the morbidostat substantially cheaper.

The construction of the morbidostat proceeded in three steps. Firstly, the construction of the morbidostat culture vials. Secondly, the construction of the vial holder array and its optical detection system. These two parts were placed on a magnetic stirrer in an incubator, providing the growing conditions of *N. gonorrhoeae*: an atmosphere of 6% CO _2_ and a temperature of 36°C. Finally, each part was connected with silicone tubes and computer controlled peristaltic pumps for liquid transfer ^13^.

***(i) Morbidostat culture vials.*** The morbidostat culture vial was a flat-bottom glass vial with a volume of 40 mL (Chemglass, CG-4902-08). It was closed at the top by an open-top GPI cap (Chemglass, CV-3750-0024) containing a Teflon insert with four holes (Euro-scientific). These holes were used for liquid injections (medium and antibiotics), waste extraction and for filtered air intake. Therefore, four pieces of PolyEtherEtherKetone (PEEK) tubing (BGB, 211609-25) were placed through the holes of the Teflon insert ^16^. The PEEK tubing ends were connected to high temperature-resistant silicone tubing (Carl Roth, 9556.1) on the outside of the culture vial. Different parts of silicone tubing were connected using Luer connectors (male and female) (Nordson Medical, MTLL004-6005 and FTLL004-6005). Each culture vial contained a magnetic stirrer bar (Carl Roth, PK75.1), to maintain a constant stirring of the culture. All materials used for assembling, were temperature-resistant and autoclavable ^13^.

**Figure 3.**
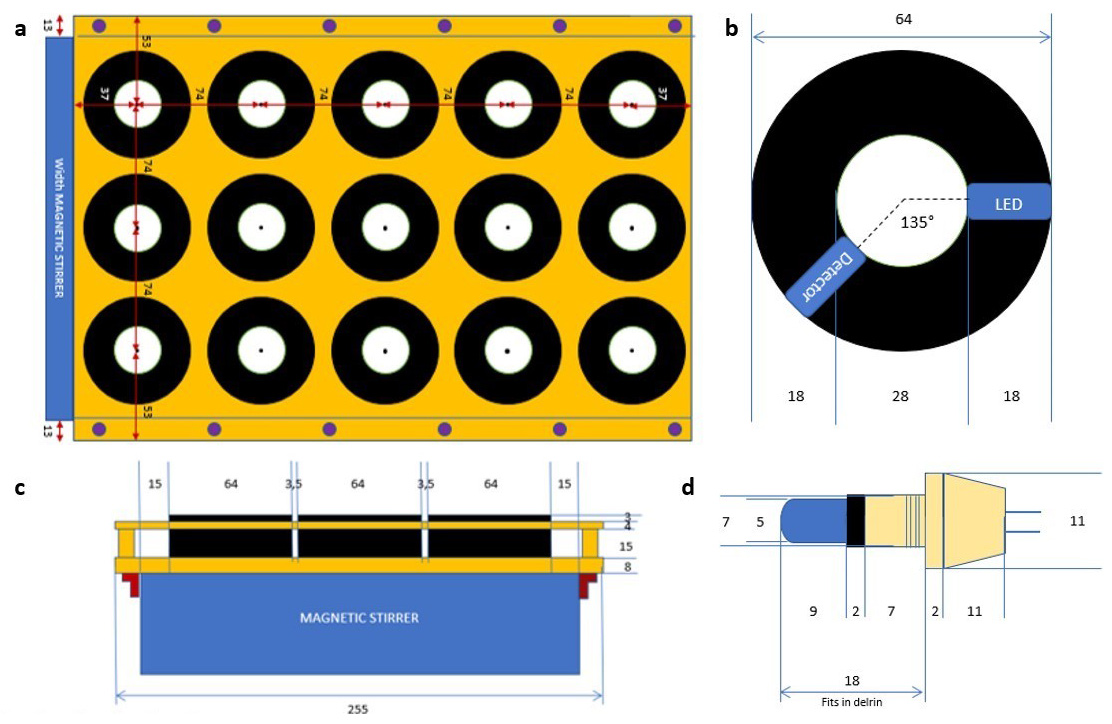
Dimensions of the vial holder array. ( **a**) Top view of the vial holder array sitting on the magnetic stirrer plate, ( **b**) Top view of the Delrin ring with the light emitting diode (LED) and detector, ( **c**) Side view of the vial holder array which sits on the magnetic stirrer plate and ( **d**) the dimensions of the LED/detector used.

***(ii) Vial holder array and its optical detection system.*** When the morbidostat was in operation mode, the morbidostat culture vials were placed in the holder array, a figure of the vial holder array with all its dimensions is shown in Figure 3. In the tube holder, infrared (IR) light emitting diodes (LEDs) (Velleman, L-7113E3BT) and photodetectors (Velleman, L-7113P3C) were machined in a black Delrin material by two openings, positioned at an angle of 135° to maximize scattered light detection. The LEDs and photodetectors were the optical detection system, and measured the optical density or turbidity in the morbidostat culture vials at set time intervals. The LEDs were connected to a relay-interface device, that could switch ON/OFF to a voltage of approximately 6V. The photo-detectors were connected with a data acquisition device (DAQ) card (Measurement Computing, USB-1616FS). This DAQ card recorded the analog voltage readings across the photo-detectors and sent a digital signal to the computer. The vial holder array sat on top of the 15-position magnetic stirrer (Carl Roth, EHY9.1) that ensured continuously stirring at a speed of 200 rotations per minute (rpm) in each culture vial ^13^.

***(iii) Assembling of the controlled peristaltic pump array.*** The last part of the construction was the connection of silicone tubes and computer controlled peristaltic pumps for liquid transfer to/from the morbidostat culture vials. All different parts with silicone tubes were connected to each other using Luer connectors. These silicone tubes exited the culture vials via the incubator (Memmert, ICO150med) through a hole at the backside. The 30 peristaltic pumps and the 16-channel suction pump were controlled with a relay interface device (Measurement Computing, USB-ERB08 and USB-ERB24), that is connected to a computer. The pumps were computer controlled using an algorithm coded in MATLAB software (MATLAB R2015b). The control software for the NG morbidostat is available from GitHub (see Software availability). The whole code was based on the code Toprak *et al*. used ^13^. The code ‘ *ExperimentController.m’* was the main class file, which ran the whole experiment by controlling *‘PumpController.m’*, *‘DataReceiver.m’* and ‘ *DataMonitor.m’* in parallel. ‘ *PumpController.m’* controlled the 30 peristaltic pumps and the 16-channel suction pump. The file ‘ *DataReceiver.m*’ contained the code that read the voltage measurements by the photo-detectors. It converted the voltage readings automatically to turbidity measurements, depending on the calibration parameters. The file ‘ *DataMonitor.m’* monitored the real-time data to the user while the experiment was running. ** Algorithm parameters like dilution time, growth time and mixing time could be set for the experiment. The 16-channel peristaltic pump was used to remove the waste/excess volume in the morbidostat culture vials.

To make sure the whole system was sterile, all tubing was autoclaved at 121°C for 21 minutes followed by a washing cycle with bleach (7%), sterile water, ethanol (70%), sterile water and growth medium, respectively, before use ^13^.

### Proof-of-concept

**Figure 4.**
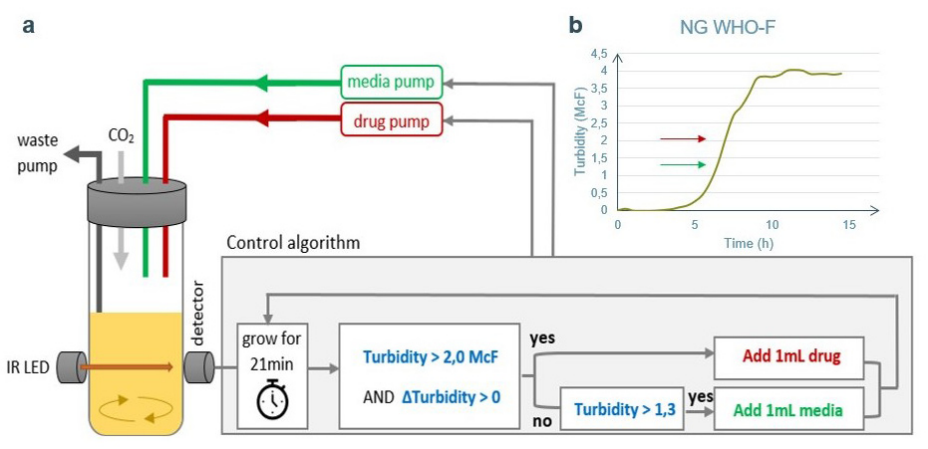
( **a**) Control algorithm used in the NG morbidostat. Thresholds used in this algorithm were based on values obtained from the ( **b**) growth curve of *N. gonorrhoeae,* green and red arrows at the mid-log phase correspond with the thresholds for medium and antibiotic injections, respectively.

**Figure 5.**
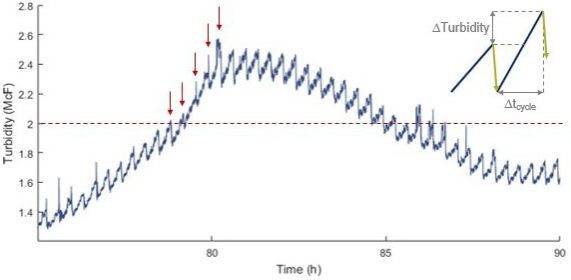
Representative bacterial growth in the NG morbidostat. Red arrows indicate where antibiotics were added to the culture. Every small peak in this curve corresponds to a cycle of 21 minutes.

We used an inoculation volume of 10 µL from a *N. gonorrhoeae* bacterial suspension (WHO-F and WHO-X) of 4.0 McF in 12 mL GC Broth supplemented with 1% IsoVitaleX (BD BBL™) enrichment for each morbidostat culture vial. All culture vials were autoclaved at 121°C for 20 minutes before use. *N. gonorrhoeae* grew in the morbidostat in cycles of 21 minutes and after each cycle, depending on turbidity measurements and growth rate, an algorithm in the software diluted the suspension with 1 mL fresh medium or with 1 mL fresh medium containing antibiotics. We set 1.3 McF as a threshold for addition of fresh medium, to allow *N. gonorrhoeae* to adapt to the environment without being diluted. Fresh medium with antibiotic was injected when a threshold of 2.0 McF was exceeded and the net growth was positive, otherwise fresh medium was injected. The algorithm used, is shown in Figure 4. After 800 seconds, a 16-channel suction pump removed the excess liquid. We took samples every 2–3 days by disconnecting the culture vials and swiping an inoculating loop through the suspension. Samples were then grown on blood agar plates and after approximately 24 hours, we stored these cultures from the blood agar plates into skim milk at -80°C ^14^. Azithromycin susceptibility testing was performed using E-tests (Biomerieux). E-tests were performed on GC-agar plates and ATCC strain 49226 and strain WHO-X were used for quality control. In Figure 5 the influence of bacterial growth when adding antibiotic is shown.

## Results & discussion

### Optimization

*N. gonorrhoeae* is very sensitive to environmental changes and requires a nutrient rich growth medium, we encountered a few issues during the optimization of our NG morbidostat.

***i. Temperature optimization.*** After the incubator was set and validated on a temperature of 36.5°C, we were not able to maintain proper growth at each position of the morbidostat. A particularly problematic position was the middle part of the vial holder. Temperature measurements using temperature probes inside the morbidostat culture vials, revealed temperatures ranging from 38°C to 41°C, which is too hot for optimal growth of *N. gonorrhoeae*. To solve this problem, we had to remove the upper plexi plate of the vial holder to maintain a homogenous and adequate temperature distribution between the culture vials. In addition, we changed the setpoint temperature in the incubator itself to 35°C, as the motor of the magnetic stirrer plate generates heat in its direct environment and *N. gonorrhoeae* is very sensitive to these environmental changes. With these adjustments, temperatures measured in the morbidostat culture vials were in the right temperature range (approximately 36°C) for all 15 vials.

***ii. Contamination.*** Compared to previous morbidostat experiments described in literature ^11, 13, 15, 17^, where a minimal growth medium is used, *N. gonorrhoeae* requires a nutrient rich growth medium (GC Broth) which in its turn increases the risk of contamination.

The most common contaminant of our culture media were *Bacillus* species. This rapidly growing contaminant resulted in two major problems. First, the nutrients available in the medium were used by these contaminants thus insufficient for the gonococcus growing in the culture vials. Second, when we used 0.2 µm filters between (a) the media reservoir and the peristaltic pump and (b) the peristaltic pump and the morbidostat culture vial, filters were clogged very rapidly. As a consequence (a) the peristaltic pumps could no longer draw the medium into the system or (b) pressure overload occurred in the tubing between the peristaltic pump and the morbidostat culture vial, causing a disconnection between the peristaltic pumps and the tubing. Therefore, we do not recommend to work with these filters when there is high risk of contamination in the culture medium. Our next step was to limit contamination by placing the medium reservoir in a UV-light-box, but in less than 24 hours contamination reoccurred. Ultimately the only way we found to prevent this contamination was to add a vancomycin, colistin, nystatin and trimethoprim selective supplement (VCNT) to the growth medium. VCNT inhibits the growth of most other micro-organisms excluding *N. gonorrhoe*ae ^18^. This step solved the contamination problem.

### Proof of concept – *in vitro* evolution of resistance to azithromycin

As proof of concept, we used the NG morbidostat to generate resistance to azithromycin. In this pilot experiment we used single vials with two strains of *N. gonorrhoeae* (WHO-F and WHO-X) exposed to increasing concentrations of azithromycin. We used the same two strains of *N. gonorrhoeae* contemporaneously exposed to growth medium only, as controls.

**Figure 6.**
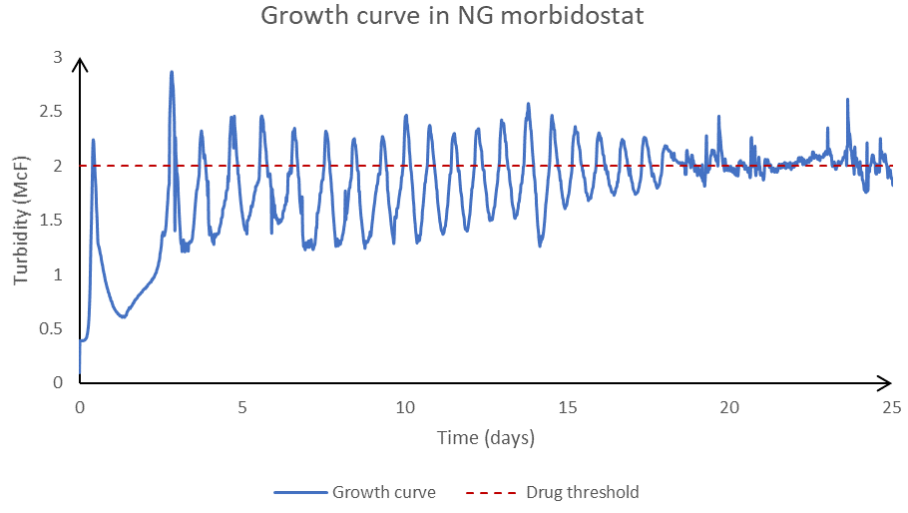
Growth curve of the population during a morbidostat experiment.

We observed the highest bactericidal rate soon after the first azithromycin exposure. After this initial exposure, the population needed a longer time to return to their log phase than later on in the experiment. Figure 6 shows a growth curve of the population during the experiment.

**Figure 7.**
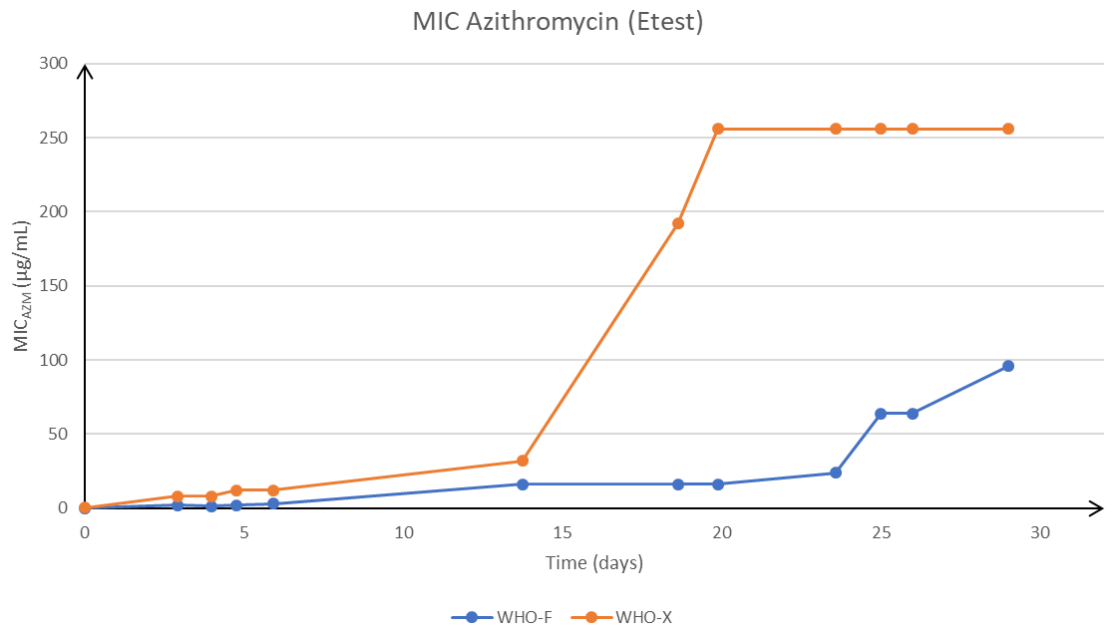
Evolution in azithromycin-resistance in morbidostat of Neisseria gonorrhoeae strain WHO-F and WHO-X over time using E-test.

The initial minimal inhibitory concentrations (MICs) of WHO-F and WHO-X were 0.125 µg/mL and 0.25 µg/mL respectively. In the first week, the MICs of WHO-F and WHO-X increased approximately 24-fold and 48-fold, respectively. By the end of the experiment (30 days), the MICs of WHO-F and WHO-X had increased more than 750-fold and 1000-fold, respectively ( Table 1; Figure 7).

**Table 1.** Minimum inhibitory concentrations (MICs) measurements in morbidostat over time, using E-test.

time (days)	MIC _AZM_ (µg/mL)	
	WHO-F (v1)	WHO-X (v6)
0	0.125	0.25
3	2	8
4	1.5	8
5	2	12
6	3	12
14	16	32
19	16	192
20	16	>256
24	24	>256
25	64	>256
26	64	>256
29	96	>256

## Conclusion

In conclusion, we described how to adapt the morbidostat device developed by Toprak *et al.* ^13^ to a version that is able to maintain *N. gonorrhoeae* growth ** under constant antibiotic pressure. The key adaptations involved building the morbidostat within a CO _2_ incubator, fine-tuning the positioning of the vial holder array and the use of VCNT. This enabled us to induce high level azithromycin resistance in *N. gonorrhoeae* within 30 days. We plan to repeat these experiments for azithromycin and ceftriaxone in triplicate and perform whole-genome sequencing on those samples to characterize the sequential mutation steps in the resistance genesis. In future experiments we also plan to use the NG Morbidostat to evaluate how different antimicrobial combinations (including antiseptic products ^19^ and bacteriophages) may be used to prevent the emergence of antimicrobial resistance in *N. gonorrhoeae*.

## Data availability

### Underlying data

Figshare: data_proof_of_concept. https://dx.doi.org/10.6084/m9.figshare.7987169 ^20^

This project contains the following underlying data:

- data_proof_of_concept_experiment.xlsx (Proof of concept – *in vitro* evolution of resistance to azithromycin underlying data)
- raw_data_figure5.xlsx (Data underlying Figure 5)

Data are available under the terms of the Creative Commons Attribution 4.0 International license (CC-BY 4.0).

## Software availability

Source code: https://github.com/everhoeven/NG_Morbidostat

Archived source code: http://doi.org/10.5281/zenodo.2643437 ^21^

Licence: MIT
