# Supplementary material for: Construction and optimization of a ‘NG Morbidostat’ - An automated continuous-culture device for studying the pathways towards antibiotic resistance in Neisseria gonorrhoeae
Source: F1000Res. 2020 Jan 8;8:560. Originally published 2019 Apr 26. [Version 2] doi: 10.12688/f1000research.18861.2 (PMC7156024; doi:10.12688/f1000research.18861.2)
Supplement: Peer review history

# Peer review: Reviewer response for version 2

**DOI:** 10.5256/f1000research.23861.r58407

**Published:** 2020-04-09

**Authors:** Olusegun Soge

**Affiliations:** 1 Neisseria Reference Laboratory, Departments of Global Health and Medicine, University of Washington, Seattle, WA, USA

The authors have satisfactorily addressed my review comments. I recommend acceptance of the paper.

I confirm that I have read this submission and believe that I have an appropriate level of expertise to confirm that it is of an acceptable scientific standard.

# Peer review: Reviewer response for version 2

**DOI:** 10.5256/f1000research.23861.r58408

**Published:** 2020-01-09

**Authors:** William M. Shafer

**Affiliations:** 1 Department of Microbiology and Immunology, Emory University, Atlanta, GA, USA

I am satisfied with the responses and revisions and recommend acceptance.

I confirm that I have read this submission and believe that I have an appropriate level of expertise to confirm that it is of an acceptable scientific standard.

# Peer review: Reviewer response for version 1

**DOI:** 10.5256/f1000research.20670.r50219

**Published:** 2019-08-01

**Authors:** Olusegun Soge

**Affiliations:** 1 Neisseria Reference Laboratory, Departments of Global Health and Medicine, University of Washington, Seattle, WA, USA

This interesting manuscript describes the construction and optimization of the ‘NG Morbidostat’ for *in vitro* selection of antimicrobial resistance in *Neisseria gonorrhoeae*. The NG Morbidostat would be valuable for real-time investigation of the evolution of antimicrobial resistance in *N. gonorrhoeae*. I have a few comments for the authors to consider.

1. What is the rationale for selecting the two WHO strains used for the proof of concept experiment?
2. Did the authors perform species confirmatory tests on the presumptive *N. gonorrhoeae* colonies cultured over the period of the experiment (30 days)?
3. Please mention the media and QC strains used for Etest.
4. I would recommend performing agar dilution antimicrobial susceptibility testing with a standard panel of antimicrobials to confirm that there was no cross-resistance to other antimicrobials.
5. It has been described previously that high-level azithromycin resistance can develop rapidly in the laboratory in *N. gonorrhoeae* isolates with a single mutated 23S rRNA allele. ^1^ What are the genetic and phenotypic characteristics of the two WHO strains? Could the presence of multiple resistance-conferring mutations, including a single-base-pair deletion within a 13-bp inverted-repeat sequence in the *mtrR *promoter of WHO X ^2^ have contributed to the higher azithromycin MICs obtained when compared to WHO F?

I confirm that I have read this submission and believe that I have an appropriate level of expertise to confirm that it is of an acceptable scientific standard, however I have significant reservations, as outlined above.

## References

[1] : High-level azithromycin resistance occurs in Neisseria gonorrhoeae as a result of a single point mutation in the 23S rRNA genes. *Antimicrob Agents Chemother*.2010;54(9) : 10.1128/AAC.00309-10 3812-6 10.1128/AAC.00309-10 20585125PMC2935028

[2] : The novel 2016 WHO Neisseria gonorrhoeae reference strains for global quality assurance of laboratory investigations: phenotypic, genetic and reference genome characterization. *J Antimicrob Chemother*.71(11) : 10.1093/jac/dkw288 3096-3108 10.1093/jac/dkw288 27432602PMC5079299

# Response

**Published:** 2019-09-13

**Authors:** Jolein Laumen

**Affiliations:** Institute of Tropical Medicine, Belgium

We would like to thank the reviewers for their valuable comments. Please find our responses below:

**1.** **What is the rationale for selecting the two WHO strains used for the proof of concept experiment?**

 For the proof of concept experiment we chose WHO-F because this strain is susceptible to the major relevant anti-gonococcal antibiotics (penicillin G, tetracycline, ciprofloxacin, azithromycin, cefixime, ceftriaxone and spectinomycin) ^1^. It thus enables us to study the evolution of AMR from a wildtype strain. The WHO-X strain was chosen because it is already resistant to multiple antibiotics, including penicillin G, tetracycline, ciprofloxacin, ceftriaxone (MIC 2) and cefixime (MIC 4) ^1^. WHO-X also has the 13 bp inverted repeat in its mtrR promoter and has a slightly elevated azithromycin MIC (0.25). These features may enhance the development of resistance to azithromycin faster or via a different pathway.

**2.** **Did the authors perform species confirmatory tests on the presumptive *N. gonorrhoeae *colonies cultured over the period of the experiment (30 days)?**

 The presumptive *N. gonorrhoeae*colonies of the proof of concept experiment were not confirmed by additional testing. However, in a soon to be published detailed follow-up experiment we performed whole genome sequencing of the obtained samples that confirmed the identity of the isolates as *N. gonorrhoeae.*

**3.** **Please mention the media and QC strains used for Etest.**

 E-tests were performed on GC-agar plates and ATCC strain 49226 and strain WHO-X were used for quality control.  

**4.** **I would recommend performing agar dilution antimicrobial susceptibility testing with a standard panel of antimicrobials to confirm that there was no cross-resistance to other antimicrobials.**

 Thank you for this useful comment. We have just finished a more detailed follow-up experiment wherein we will report the antimicrobial susceptibility of different antimicrobials (tested via agar dilution). 

**5.** **It has been described previously that high-level azithromycin resistance can develop rapidly in the laboratory in *N. gonorrhoeae* isolates with a single mutated 23S rRNA allele.** **^1^** **What are the genetic and phenotypic characteristics of the two WHO strains? Could the presence of multiple resistance-conferring mutations, including a single-base-pair deletion within a 13-bp inverted-repeat sequence in the *mtrR *promoter of WHO X** **^2^** **have contributed to the higher azithromycin MICs obtained when compared to WHO F?**

 The WHO-F strain has a wild type genetic profile whereas the WHO-X strain contains various mutants, including the 13-bp inverted repeat sequence in the *mtrR*promoter. The WHO-X strain does not have any mutations in the 23S rRNA gene. In our more detailed follow-up experiment we saw that the WHO-F strain also reached a MIC of >256 (determined by E-test). However, as in the current paper, the WHO-X strain developed high level resistance faster than WHO-F. This faster acquisition could be due to the genetic profile of WHO-X. Whole genome sequence analysis of this follow-up experiment will be described in a subsequent paper.  

 References      

1. Unemo M, Golparian D, Sánchez-Busó L, Grad Y, Jacobsson S, Ohnishi M, Lahra MM, Limnios A, Sikora AE, Wi T, Harris SR. The novel 2016 WHO Neisseria gonorrhoeae reference strains for global quality assurance of laboratory investigations: phenotypic, genetic and reference genome characterization. Journal of Antimicrobial Chemotherapy. 2016 Jul 17;71(11):3096-108.

# Peer review: Reviewer response for version 1

**DOI:** 10.5256/f1000research.20670.r50218

**Published:** 2019-06-25

**Authors:** William M. Shafer

**Affiliations:** 1 Department of Microbiology and Immunology, Emory University, Atlanta, GA, USA

This manuscript is an important contribution to the field of antibiotic resistance development in *Neisseria gonorrhoeae*. The "NG Morbidostat" detailed by the authors has many advantages for studies on evolution of antibiotic resistance in culture. The construction and use of the system is well described. I have a few technical questions that require the attention of the authors:

1. Please indicate the colony type of the two strains employed (state of piliation and opacity).
2. For azithromycin, Etest strips can give variable results so it is important to verify the deduced MIC by the more accepted agar dilution method. Also, was the Etest assay performed on outgrowth of single colonies or the batch culture? Did the authors check single colonies?
3. It is not clear as to why blood agar plates were employed as opposed to the more conventional GC agar? 
4. It would seem that the progression in azithromycin MICs represented initial mutations in the mtr locus followed by 23S rRNA genes, This could be checked quickly by PCR/sequencing.

I confirm that I have read this submission and believe that I have an appropriate level of expertise to confirm that it is of an acceptable scientific standard, however I have significant reservations, as outlined above.

# Response

**Published:** 2019-09-13

**Authors:** Jolein Laumen

**Affiliations:** Institute of Tropical Medicine, Belgium

We would like to thank the reviewers for their valuable comments. Please find our responses below:

**1.** **Please indicate the colony type of the two strains employed (state of piliation and opacity).**

This article describes the construction and optimization of the NG morbidostat. Its primary goal is to share how we constructed/optimized the morbidostat, and in particular how we overcame stumbling-blocks along the way. We thought this would be a useful contribution to the field as it may help other groups who may wish to construct in-vitro models to test various theories about gonococcal AMR selection. The article also includes a proof of concept experiment. We have recently completed larger scale experiments where we have conducted the various steps suggested by the reviewers such as describing the colony type, confirming MIC results with agar dilution and performed whole genome sequencing of the isolates every 3 to 5 days. These results will be described in a forthcoming paper.

**2.** **For azithromycin, Etest strips can give variable results so it is important to verify the deduced MIC by the more accepted agar dilution method. Also, was the Etest assay performed on outgrowth of single colonies or the batch culture? Did the authors check single colonies?**

Because this was a proof of concept experiment, we performed E-tests on the batch culture. We have previously compared the results of azithromycin E-tests with agar dilution in our laboratory and found them to be comparable, as was also found by others (Liu H, Taylor TH, Pettus K, Trees D. Assessment of Etest as an alternative to agar dilution for antimicrobial susceptibility testing of Neisseria gonorrhoeae. Journal of clinical microbiology. 2014 May 1;52(5):1435-40). 

**3.** **It is not clear as to why blood agar plates were employed as opposed to the more conventional GC agar?**

We used blood agar plates instead of GC agar plates because this allowed us to check for a broader spectrum of contaminants. 

4. **It would seem that the progression in azithromycin MICs represented initial mutations in the mtr locus followed by 23S rRNA genes. This could be checked quickly by PCR/sequencing.**

The progression in azithromycin MIC indeed suggests initial mutations in the mtr locus followed by 23S rRNA genes. We are currently analyzing the whole genome sequencing data from our larger follow-up experiment and will then be able to provide this information in a subsequent paper.
